# The experiences of men following recurrent miscarriage in an Irish tertiary hospital: A qualitative analysis

**DOI:** 10.1111/hex.13452

**Published:** 2022-03-03

**Authors:** Tommy Harty, Maria Trench, Orla Keegan, Keelin O'Donoghue, Daniel Nuzum

**Affiliations:** ^1^ Department of Medicine Cork University Hospital Cork Ireland; ^2^ Pregnancy Loss Research Group, Department of Obstetrics and Gynaecology University College Cork Cork Ireland; ^3^ Graduate School of Healthcare Management Royal College of Surgeons Ireland Dublin Ireland; ^4^ Department of Education and Bereavement Irish Hospice Foundation Dublin Ireland; ^5^ Department of Obstetrics and Gynaecology Cork University Maternity Hospital Cork Ireland; ^6^ The Irish Centre for Maternal and Child Health Research (INFANT) University College Cork Cork Ireland

**Keywords:** fathers, men, miscarriage, qualitative, recurrent miscarriage, users' experiences

## Abstract

**Introduction:**

Miscarriage is one of the most common complications of pregnancy, and recurrent miscarriage affects approximately 1% of couples. The psychological impact of early pregnancy loss on women has been well documented in the literature; however, the burden of miscarriage on men remains largely unexplored.

**Methods:**

This qualitative research involved semi‐structured interviews with five men whose partners had experienced at least two consecutive miscarriages. Participants were recruited through an early pregnancy loss clinic in a large, tertiary maternity hospital. Interviews were recorded and transcribed verbatim and analysed thematically.

**Results:**

Recurrent miscarriage had a pronounced psychological impact on all the men interviewed, which worsened with each successive miscarriage. Three primary themes were developed from the data: (1) the deeply emotional experiences of men following recurrent miscarriage; (2) frustrations experienced during the provision of support following recurrent miscarriage; and (3) a sense of feeling unimportant. Lack of timely provision of information about miscarriage as well as lack of access to services were highlighted as deficiencies in the quality of care provided after recurrent miscarriage.

**Conclusion:**

The experiences of men after recurrent miscarriage are based largely on their assumed role as the protector and supporter of their partner, which often results in neglect of their own psychological needs. The support required by men is similar to that required by women, and greater access to information and services is needed to improve the experiences of men following recurrent miscarriage.

**Patient Contribution:**

Participants were recruited through the Pregnancy Loss Clinic at Cork University Maternity Hospital and were identified by specialist midwives. Participants were approached and interviewed by one of the researchers. Participation was voluntary and the men received no financial contribution for their time.

## 1 INTRODUCTION

Miscarriage is one of the most common complications of pregnancy, and in Ireland, it is defined as the loss of pregnancy before 24 weeks of gestation.^1^ Recurrent miscarriage, where two or more consecutive pregnancies end in loss, affects approximately 1% of the reproductive‐age population.^2^, ^3^, ^4^ The need for familial and social support has been highlighted previously, given the deeply emotional and distressing experiences that can be associated with pregnancy loss.^5^, ^6^ The often profound emotional distress associated with recurrent miscarriage is experienced by both the couple as a unit as well as by partners individually. An extensive body of research has explored the psychological burden as well as the health and well‐being outcomes of women who have experienced miscarriage,^4^, ^7^, ^8^, ^9^ and in recent years, some academic interest has been demonstrated regarding the impact that pregnancy loss has on male partners.^5^, ^10^ However, the experiences of men, particularly in the setting of recurrent miscarriage, remain largely underevaluated.

A heightened awareness of the psychological impact that pregnancy loss has on bereaved parents has led to increased research interest in this area in recent years.^11^, ^12^ Parents who have experienced a miscarriage often feel an overwhelming sense of grief that is coupled with a sense of loneliness in coping with this loss, as they are often excluded from the normal grieving processes that would occur in other cases of death that are considered less ambiguous and more legitimate.^13^ Such experiences can lead to a disenfranchized experience of loss, a maladaptive grieving and bereavement process and may incur strain on familial relationships.^14^ Evaluation of the psychological morbidity associated with miscarriage in women has led to the development of a number of supports to address their needs.^15^ However, few interventions are tailored specifically for men despite the potentially negative impact of the experience on their well‐being.

It has been reported that although men show different response patterns after the event of a miscarriage, in general, the emotions experienced are similar to those felt by women: grief, anxiety, stress and depression.^10^ Some quantitative data suggest that men are just as likely to experience as high or higher levels of grief as their female partners^16^; however, following pregnancy loss, men are more likely to engage in active‐avoidance coping mechanisms and resulting maladaptive compensatory behaviours such as smoking and increased alcohol consumption.^10^, ^17^ Men are more likely to internalize their emotions, which may be attributable to the lack of recognition of their loss as well as the societal perception and pressure on men to be strong and supportive of their partners during a time of significant loss.^10^ Men who internalize these grief emotions are less likely to engage with or seek out health services, which can impact negatively on their intimate relationships.^18^ When men were perceived by their partners to be open about their feelings and engaging with caring behaviours, they were less likely to experience negative outcomes in their intimate relationships than those who were not.^18^

The need for support following miscarriage for men has been acknowledged and the literature to date emphasizes that men are deeply emotionally affected by their loss of role as a father, manifesting as grief and frustration.^5^ Due et al.^10^ highlight that the hospital experience at the time of miscarriage diagnosis is a crucial source of support for couples and opens the discussion for future support services that are available. Support services for men have increased in recent years, and pregnancy loss organizations such as the Miscarriage Association in the United Kingdom and SANDS, Australia, specifically recognize the impact that miscarriage can have on men and families.^19^, ^20^ Information leaflets on the Miscarriage Association website provide detailed and supportive information for men, acknowledging the feeling of loss as a father and addressing strategies that men can use to cope with this loss and how miscarriage may affect relationships and fertility in the future.^19^ Further details of where men can find additional help and support are also available from these organizations. However, in Ireland, at the time of this study, there were no specific support structures or services for men following miscarriage.

Recurrent miscarriage presents as a unique health challenge in terms of managing the emotional morbidity felt by both men and women. A recent study by Koert et al.^21^ was the first to address the needs of couples experiencing recurrent pregnancy loss and highlighted the apparent disconnect between the couple's needs and their experience of medical care after recurrent miscarriage. Furthermore, these authors suggest that the isolated examination of the experiences of men is grossly unexplored.

The aim of this paper is to address a gap in the current literature by analysing, through interviews, the experiences of men following their partner's recurrent first‐trimester miscarriage. Following this, we hope to determine the necessary supports and/or interventions that are required to support men in their bereavement and grief journey.

## 2 METHODS

Qualitative methods were used in this study. Qualitative research can be used to better understand complex social processes, to capture essential aspects of a phenomenon from the perspective of study participants, and to uncover beliefs, values and motivations towards the quality of care and service provision.^22^, ^23^ Qualitative methods have previously been used extensively to evaluate outcomes in the field of pregnancy loss and effectively capture the lived experiences of women and men after miscarriage and pregnancy loss.^5^, ^9^, ^24^, ^25^ This helps to inform clinical practice with regard to the identification of deficiencies in the quality of care provided and how, as clinicians, we can better improve the experiences of couples and families at a particularly distressing time in their lives. Qualitative methods can effectively capture patterns of meaning from the richness of data that are required to best understand the experiences of men affected by recurrent miscarriage.

### 2.1 Ethical approval

Ethical approval was sought and granted from the Clinical Research Ethics Committee of the Cork Teaching Hospitals (CREC) on 9 February 2019. A distress protocol was in place should any participant become overtly upset during the interview process; however, this was not required.

### 2.2 Recruitment

Men were recruited through a pregnancy loss clinic in an Irish tertiary maternity hospital. A purposive sample of nine men was approached by the specialist midwives in bereavement and loss with an invitation to participate in the study. Inclusion criteria were that the participants were partners of women who had been cared for in the study hospital and had experienced two or more first trimester miscarriages, were over 18 years old and had not previously indicated that they did not wish to be contacted by the hospital for study purposes. Men were excluded if they did not have English as their first spoken language. The rationale for recurrent first trimester miscarriage was informed by the literature and the lack of focus on this particular cohort.

Once deemed eligible for the study, men were issued with an invitation to participate in the study and asked to contact the primary interviewer, following which an appointment for a semi‐structured interview was made to take place at the study hospital.

Semi‐structured interviews were conducted by one of the researchers. Semi‐structured interviews ensure uniformity in the content of the interview, while also providing an additional opportunity to capture the lived experienced of the individual's experience.^26^ The interview process was supported by two specialist midwives in bereavement and loss with experience in the provision of care to couples who experience pregnancy loss, whose purpose in this study was to identify men from the pregnancy loss clinic and refer them to the interviewer. The specialist midwives were not present during the interview. However, they were available as part of the distress protocol, should it be required.

### 2.3 Data collection

A semi‐structured interview guide with open questions was developed by the authors based on a comprehensive review of the literature^10^, ^21^, ^27^, ^28^, ^29^ and the authors' professional knowledge and experience working in a perinatal bereavement specialist team. The interview schedule included a series of open‐ended questions pertaining to the participants' experiences of the miscarriage, their opinion of the service provided by the relevant healthcare professionals, their feelings towards their baby and partner and their experience of the support available to them, as men, following the diagnosis of miscarriage. Following written consent, interviews with the men took place in a private environment within the study hospital. Audio recordings of the interviews were transcribed for analysis purposes. Transcriptions were anonymized for confidentiality purposes and checked by the researcher, M. T., before analysis to ensure accuracy of the data.

### 2.4 Data analysis

Phenomenological methods were used in this study. The aim of phenomenology is to describe the meaning of an experience, both in terms of what happened and how it was experienced.^30^ Using this method, we provide detailed examinations of the men's lived experiences in their own terms rather than a predetermined theoretical approach, with the aim of understanding the depth of experience of each man following recurrent miscarriage, before making more generalized claims. The data generated from the interview process were analysed using a close interpretive reading of the transcripts, and notes were translated into themes using an iterative thematic approach to provide a rich understanding of the phenomenon of recurrent miscarriage for men.^31^

Each transcript was reviewed by the researcher, M. T., multiple times. Inductive methods were used to develop thematic patterns from the data to capture the meanings associated with recurrent miscarriage experiences for the participants. These themes were coded accordingly. A coding framework was developed, and coded text was collated and analysed for similarities and differences. The data were handled and analysed manually. The researcher was aware of the importance of self‐reflexivity in the process of engaging with the data, recognizing areas of personal identification with the stories of participants, areas of divergence and the interpretation of the data in the development of the themes. Of particular note was the personal experience and history of miscarriage for the researcher and an awareness of the reflexive importance of this experience alongside the experiences of participants.

Themes, defined as a piece of information that captures something important about the data in relation to the research question and represents some level of patterned response or meaning within the data set,^31^ were identified. Following this, the data were reviewed and further classified into subthemes. A second researcher, D. N., reviewed the manuscript transcriptions for accuracy, and the authors reached consensus on the final interpretation of the data.

## 3 FINDINGS

Five men consented to participate in the semi‐structured interviews. The remaining four invitees were either unavailable or declined to participate in the study. This small sample size reflects the availability of participants within this specific study population, and is comparable to similar studies,^32^, ^33^ as the challenges of recruiting men for reproductive research are acknowledged.^34^ Details pertaining to the number of miscarriages experienced by each participant are described in Table 1. Interviews were conducted between May and July 2019 and ranged in duration from 40 min to 2 h and 10 min. The mean interview time was 65 min. Direct quotes are used in this paper to support the results and represent the themes identified in the interviews.

**Table 1:** Participant characteristics

Participant number	Number of miscarriages experienced	Living children at the time of the interview
1	4	2 (Both children from a previous relationship)
2	3	0
3	3	0
4	4	2
5	3	2

Three main themes were developed from the data set: (1) the deeply emotional experiences of men following recurrent miscarriage; (2) frustrations experienced during the provision of support following recurrent miscarriage; and (3) a sense of feeling unimportant. Subtheme analysis was performed addressing specific points related to each overall theme.

*Theme 1: The deeply emotional experiences of men following recurrent miscarriage*

1. *Projection of joy and excitement for the future associated with the news of the first pregnancy*
  All the men detailed their initial happiness regarding the news of the first pregnancy and how their first, immediate thought was that of the future and the child that their partner would bring into the world. None of the men initially thought that the pregnancy may evolve into a significantly different outcome. That feeling of joy and excitement was diminished with each subsequent pregnancy, as the men became familiar with a sense of loss associated with the failure of a previous pregnancy.
*You are looking forward to the next nine months……and ……. Yes, the child will appear in nine months' time or whatever and you are thinking of baby names and stuff like that*. (Participant 1)
*So we were very excited, yes……You kind of suddenly start adjusting your life to thinking that in nine months everything is going to be different, so you're just trying to plan everything, you're thinking changes quite a lot in terms of what you are doing ………so yes, we were very excited*. (Participant 3)
  In the setting of recurrent miscarriage, this feeling of excited anticipation regarding the future and the men's potential roles as new fathers was shown only upon news of the first pregnancy and was lost in subsequent pregnancies.
2. *Deep connection and protective response evoked after visualization of ultrasound imaging*
  A strong and recurrent theme in the data was the impact that viewing the ultrasound image of the pregnancy had on each of the men. Visualization of the ultrasound images prompted a deep emotional response in the men and heightened the men's connection with their child. In particular, greater emotional connections were observed in men after hearing and seeing the foetal heartbeat.
*Like when you got the heartbeat, that's amazing. That changes everything*. (Participant 5)
  The above excerpts from three of the interviewed men's experiences of viewing the ultrasound images demonstrate how this helps to establish a deep emotional connection with their unborn child, which is ultimately destroyed following their loss after miscarriage.
*The second time was worse because we had this little picture, a heart was beating*. (Participant 1)
*Yes, you could see……. Yes…… It's like you know the hairs raising on your hand kind of thing …you'd be excited to see it …….especially for the likes of us when there would have been a few misses ………….maybe you know, one in every three might come good or something to that effect ……*. (Participant 4).
  It is clear from these excerpts that visualizing the foetal heartbeat offers an overwhelming sense of hope to these men that this pregnancy may be the one that is successful, a feeling that has been built on the previous experience of pregnancies ending in miscarriage. Furthermore, Participant 1 highlighted that the feeling of loss with their second miscarriage was far more profound, having been offered this apparent sense of hope after seeing their child's beating heart, a phenomenon that had not occurred during this couple's first pregnancy.
*You could see a tiny…….little circle ………it made it more connected actually…….the nurse asked when we were leaving if we find anything do you want us to contact you or will we bury it and like neither myself or [partner] are religious so my initial reaction was no, we will take care of it, which made me feel a bit more connected to it then*. (Participant 3)
  Here, Participant 3 is also referring to burying foetal tissue that may be identified on laboratory analysis of the miscarried products of conception. Having something physical to hold onto and grieve by burying clearly promotes a deep emotional response in this man.
3. *Perceived role as protector for their partners following miscarriage(s)*
  All men addressed their role in supporting their partner after miscarriage. It was clear from each interview that the men viewed this as an essential role to support and protect their partners through the miscarriage process and, in some sense, to ‘fix’ the situation. One man felt the need for himself to be ‘*calm*’ to ‘*keep it together*’ for his partner (Participant 1).

*Focused on making sure she was ok, putting the arm around her……. you know what I mean, there would be stuff you are carrying ……. sure that's my role anyway, I have signed the contract …….so I, I support her through this, you know*. (Participant 2)

*I just felt like I had to step up more, to kind of be maybe more there for her and the kids*. (Participant 4)

It is noteworthy that none of the men mentioned the need to support themselves emotionally; their primary focus was to support their partners. Participant 3 described it as follows:

*It was quite scary [partner] going down for the procedure………. I was like, you know in my head…….in your head you are trying to support her*. (Participant 3)

Despite his own fears and concerns about what his partner was going through, Participant 5's primary worry was how he was going to adopt the role of supporter for his partner during this time of need.

Notably, it became more difficult for the men to maintain this role as supporter for their partners with each successive loss. One of the men in this study spoke of the cumulative nature of the losses and how, with each miscarriage, remaining positive and protective of their partner became increasingly difficult.

*Theme 2: Frustrations experienced during the provision of support following recurrent miscarriage*

Some men identified the specific support provided by the specialist midwives in bereavement and loss as the only beneficial support that they experienced following their partners' recurrent miscarriages. Overall, the quality of this service was deemed to be excellent. Admittedly, these men did not meet with the specialist midwives until after they had already experienced one miscarriage, and in some cases, it was not until after several miscarriages that the men met with this specialist support service. Furthermore, meetings with the specialist pregnancy loss team and midwives were aimed at the couple as a single unit, and not at the man individually. Some of the participants mentioned that their partners were offered information about the availability of miscarriage support groups; however, they admitted that none of the same support was offered to them individually, as men.

1. *Lack of information about the miscarriage and subsequent procedures*
  In some cases, the men emphasized the paucity of useful information that was provided to them and their partners at the time of miscarriage and thereafter. During some of the interviews, the men described how accurate information provided at the correct time could have helped alleviate much of the distress experienced across many aspects of the miscarriage experience, as well as providing clarity, hope and assurance at this time.
*Information would have been in that moment, I think, yes…………well, it made the second and third one much different experience for us definitely. Sad experiences but not as kind of, not a sucker punch in terms of getting completely thrown back*. (Participant 3)
*But that tiny little bit of advice I think would have made the world of difference……. I kind of got a bit angry after that I didn't know it was such a high percentage, because I felt I went through an awful lot of thinking it was whatever……….the chances of this*. (Participant 3)
  The provision of timely and specific information may have offered significant support for some men. The relevance of this information is particularly significant for these men who are experiencing recurrent miscarriage, whereby provision of information regarding miscarriages may have positively impacted their experience in subsequent miscarriages.
*It wasn't until we spoke to the doctor ………I didn't realise how many pregnancies go to miscarriage so, until then in my head we were one in a thousand……. how did this happen?* (Participant 3)
*Information would have been in that moment, I think, yes…………well, it made the second and third one much different experience for us definitely. Sad experiences but not as kind of, not a sucker punch in terms of getting completely thrown back*. (Participant 3)
  Participant 3 makes direct reference here to how the provision of appropriate information in previous pregnancies would have made significant difference to their experience of subsequent pregnancy losses. Having a clearer understanding of the incidence of miscarriage and why miscarriage occurs for some couples can provide support to men and their partners experiencing early pregnancy loss. It may also help attenuate the sense of isolation and loss experienced by some men following miscarriage.
2. *Practical and logistical aspects of the service provided that influenced their experience*
  The majority of the men expressed frustration at logistical aspects of the service provision in this tertiary maternity hospital. Grievances were voiced about some of the practicalities relating to the service and how, at times, it impacted negatively on their experience.
  The men felt unsupported as a result of challenges that were inherent to the system in which the service was provided.

*Pretty angry about that, yes, because like, of all the numbers if you need to be able to talk to someone ……if you are ringing a maternity line that is one thing where you really need to get an answer. You know it's not for ……you've got a runny nose or something like. You need to talk to someone*. (Participant 3)

Specifically, the men were dissatisfied with the waiting times at clinics and in the emergency rooms, which they felt was to excess, as well as not being able to contact the hospital using the given emergency telephone number when urgently required.

*Rang the number for the hospital, no answer, rang the emergency number, no answer…….so in the car……. got back up here about 8.30 in the evening. We were just waiting in a room……. while we were waiting, she was constantly bleeding so…. her pants at this stage were ruined and by the time we got into the doctor, it was 11.30 or 12 that night*. (Participant 1)

*The not knowing and just sitting there and waiting and watching the clock and there is nothing you can do. So all the times we have been to the A & E, it's been the same pattern…….A new person comes every 45 minutes or so but the time it actually takes you to go through is six to nine hours….I understand that the doctor is needed in other places but this is a dedicated emergency department for a maternity hospital*. (Participant 1)

Overall, however, they were generally satisfied with all healthcare individuals involved.

*Theme 3: A sense of feeling unimportant*

1. *Feeling unimportant as a man*
  Several of the men expressed ‘feeling in the way’ or not knowing what to do in the hospital. There was a strong sense of not being included at times or a lack of recognition that they were also going through this.
*Just ……just ……. just bring him with her………right, if he is sitting there and doesn't know where to go …. she doesn't know where to go and the nurse talks to her and doesn't engage with him. He feels he has to sit there until he is called*. (Participant 2)
*Like it would have been said generally in the room that, do you know, there's no heartbeat…. but no, nothing. I would never have been spoken to*.(Participant 5)
  A definite lack of engagement of these participants by staff working in the maternity services has excluded these men from belonging to part of the normal process of bereavement. Lack of acknowledgement of their loss as fathers may impact on their own grieving methods and certainly negatively impacted on their experience of how the diagnosis of miscarriage was conveyed to them.
*Like you are going through it as well but you're not because…you know. It's your loss aswell…….so it's my loss as well………and it's my grief but, I said……… Yes, that's kind of the case, the joke is always the man is the plus one like………*. (Participant 1)
*I think lads really struggle with what their role is when they come into the hospital ………like is it to shut up? Is it to hold hands ……….is it to cry with them …………is it to offer a few words in the hospital or wait until you get to the car?* (Participant 5)
  A certain lack of inclusivity was felt by the men during the miscarriage process that was compounded by their struggle in their role as supporter for their partners in the hospital. Again, this highlighted lack of engagement by healthcare staff with the male partners coupled with a sense of feeling unimportant was a consistent experience during each miscarriage. The men did not state that their frustrations increased despite this continued lack of acknowledgement by staff with each successive miscarriage, which likely reflects their primary concern relating to their role as supporter of their partner through loss.
2. *Lack of acknowledgement for grief of recurrent miscarriage*
  Several of the men discussed that as a couple, they felt under‐recognized, unimportant and as if they were just a number to some of the healthcare professionals. The men felt that the healthcare professionals involved were focused on completing their workload rather than supporting the couple with an in‐depth feeling of empathy and support. There is an obvious sense of frustration experienced by the men at the lack of acknowledgement as a couple of their loss in the setting of recurrent miscarriage.

*Oh, yes you are completely unimportant like, you're just a number like ………you're just kind of ……. like again……. a factory*. (Participant 1)

*I don't think, once the process is set, that it's all about empathy ………It's all about getting it done*. (Participant 2)

One man acknowledged the busy workload of the clinical staff at the early pregnancy clinic; however, he felt that, as a couple, they had been dismissed somewhat as a result of them not carrying a viable pregnancy. He felt as though they were not given equal attention from the medical staff compared to a couple who were carrying a viable pregnancy.

*Doctors are kind of busy ……… You're not carrying anything …….so it's not that you are irrelevant obviously but maybe a bit …………. Like if we are not pregnant and they are, they need the doctor a whole pile more………I wouldn't say unimportant but less important………less important than couples who are pregnant………* (Participant 4)

Although this participant could appreciate the clinical demand required of staff, it did not lessen the palpable sense of disregard that he and his partner felt during their experience at the hospital. Another participant even suggested that providing something as simple as an information ‘flyer’ would significantly lessen this feeling of neglect.

## 4 DISCUSSION

This study aimed to address a gap in the current literature by exploring male partners' experiences of recurrent miscarriage and their perception of support available to them during and after early pregnancy loss. Findings from this study are generally consistent with what has been reported in the available literature to date regarding men's experience of pregnancy loss; however, these qualitative results provide a unique and largely unexplored perspective of the specific experiences of men who have experienced recurrent miscarriages. Through interview, the men's experiences were explored and transformed into themes that provide a detailed insight into the emotions and processes that are felt by men who have experienced recurrent miscarriages. Deep emotional responses were shown by the men in this study. Initially, the men spoke enthusiastically and excitedly about how they felt when they heard the initial news of the pregnancy. These emotions were felt most notably following news of the first pregnancy and it would seem that as they became familiar with the feeling of loss after each successive miscarriage, these emotions of hope and excitement became blunted somewhat. Hope for the future at the beginning of each successive pregnancy was not as prominent. None of the men considered that events may change at the start of the first pregnancy and what they had foreseen as potentially being a very happy outcome became a significant traumatic event for them and their partners. Undoubtedly, this added to their shock and distress when the miscarriage occurred. Further, their identity as a future father appeared to be stolen from them repeatedly. Bowman^35^ wrote about this concept of shattered dreams and how, for many people, bereavement and shattered dreams are unavoidably linked. Recurrent miscarriage can fracture these dreams and arguably intensify the bereavement process. Men need the appropriate time and support to rebuild an alternate dream.

Additionally, visualization of an ultrasound scan intensified the emotional response felt by some men due to a heightened sense of connection with their baby. These findings are consistent with previous studies that quantitatively established that men who had viewed an ultrasound before miscarriage scored higher on grief scales.^36^ Notably, within our unique sample of men who had experienced multiple losses, some of the men interviewed had the capacity to compare experiencing loss after viewing an ultrasound image to experiencing loss without imaging. Heightened emotional responses were reported by men who had experienced miscarriage after visualizing the foetal heartbeat when compared to previous pregnancies where this had not been experienced. Having experienced a miscarriage in the past, a visual recognition of the baby offered the men a sense of hope that this pregnancy might be the ‘one’; however, this initial glimmer of hope makes their loss all the more heart‐breaking when the pregnancy ultimately ends in miscarriage. McCreight^37^ reports that the overwhelming sense of loss experienced by men after viewing antenatal ultrasounds has a powerful effect in terms of damage of men's self‐identity as fathers.

Male partners' anticipated role as supporters for the partners has been reported extensively in the literature to date.^9^, ^10^, ^32^, ^38^ All the men in this study discussed their duty to act as a supporter for their partner, and in doing so, often neglected and undervalued their own needs. This seems an unsurprising finding when masculine roles and identities are explored in a societal context.^39^ Expectations of males being the carer and the stoic partner remain prevalent. Additionally, while the men wanted to do their best in supporting their partners, there was a prevailing sense of uncertainty regarding the correct way of showing their support and a resultant feeling of helplessness. This feeling of helplessness increased with each successive pregnancy loss. One of the men in this study spoke of the cumulative nature of the losses and how, with each loss, remaining positive and protective of their partner was more difficult. Indeed, this finding alone confirms the importance of putting relevant support in place for these men in the setting of recurrent miscarriages, given the accruing associated feelings.

An important pathway through which men can access support and information is through their interactions with healthcare services and professionals. However, in keeping with the findings of previous research completed in this area, the men in this study expressed some negative opinions with regard to service provision.^32^, ^37^, ^40^, ^41^ The men reported a deficiency in the timely provision of supportive information, a lack of appreciation of their role as a father who is also grieving and their frustrations towards some aspects of the care provided throughout the miscarriage. Men often feel like they are ‘in the way’ during the miscarriage process,^42^ and the results from this study highlight a need to promote inclusivity for men and provide them with an appropriate level of understanding for them to achieve a sense of ownership of their rightful place during the grievance process.

Nearly all the men identified with the feeling of being just another number, which can be interpreted as an expression of feeling both inconsequential and belonging to part of a process. These experiences could also be compounded by the reality of disenfranchized grief for men. Notably, one man independently suggested that being given an information leaflet at the initial stage would have signalled to him that he was important and included in this situation. A lack of provision of sufficient and timely information about the miscarriage process to men and details about how to access to services was highlighted as a major shortcoming during these men's experience. Public perceptions of miscarriage can often be erroneous, and the general population can have misconstrued beliefs regarding the incidence of miscarriage and its potential causes.^43^, ^44^, ^45^ Most men interviewed felt that judicious provision of important information regarding miscarriages would have offered significant support to them as partners, particularly in the context of recurrent miscarriage. Knowledge pertaining to miscarriage incidence and aetiologies, had it been provided in a timely manner from the first miscarriage, may have provided significant support and understanding to these men during subsequent losses. This information is pivotal in the planning of a support intervention for men.

Significant frustrations were expressed by the men regarding service provision. A lack of access to emergency services, such as the emergency medical and midwifery number, at critical times during the miscarriages as well as prolonged waiting times at clinics and in the emergency department were some of the grievances voiced. Parents' perception of care at a traumatic time is shaped by the environment of care around them and has considerable impact on the grieving process.^33^ The participating men appeared to interpret these shortcomings as a failure in the provision of adequate care for themselves and their partners.

The results from this study will be used to help establish a support service for men who have experienced a miscarriage in the study hospital. This will be an adjunct service to the early pregnancy loss clinic that runs in this hospital and will aim to provide men with psychological support in their journey through bereavement and loss. The support required by men is similar to that needed for women as cited in the literature: acknowledgement of loss and provision of personalized information and follow‐up care,^46^, ^47^ which begs the following question: should we consider the ‘patient’ in the setting of recurrent miscarriage as the couple rather than the man and the woman individually? Results from a recent qualitative study by Koert et al.^21^ found that couples who had experienced recurrent pregnancy loss desired a ‘couple‐focused’ approach to medical care. This includes early access to investigations for both the man and the woman and personalized supportive and psychological care that is tailored to the couple. This concept of ‘couple‐focused’ care is particularly relevant in the setting of recurrent miscarriage, which presents unique challenges to healthcare provision, given the cumulative effect of multiple losses and the toll that this takes on both partners. The data from our study highlight the importance of identifying and responding to the needs of men as part of the ‘couple’.

Future research may extend the inclusion criteria to men who have experienced first and second trimester miscarriage to deepen our understanding of the supports required by a wider audience of men following recurrent miscarriage.

## 5 STRENGTHS AND LIMITATIONS

To our knowledge, this is the first study of its kind to specifically examine in detail the lived experiences of men following their partners' recurrent miscarriages. This study provides an in‐depth account of the experiences of men and the associated interpretations related to recurrent miscarriage for male partners. These results provide maternity care clinicians with important insights about parents experiencing recurrent miscarriage, which should be used to improve the quality of care provided for men and couples going forward.

The conduct of interviews by an interviewer who is not inherently involved with the service provision is a strength of this study. It allowed the participants to discuss in detail and with honesty some of the limitations and flaws related to the quality of care provided and service provision as the interviewer was distinguishable from clinicians who may have previously dealt with the men during the miscarriage process.

All men in this study were recruited through a pregnancy loss clinic in a single Irish tertiary maternity centre. It would be useful for future studies to analyse the experiences of men recruited from a broader range of services and locations.

The sample size reflects the acknowledged challenges of recruiting men into studies in pregnancy loss.^34^, ^48^ However, the data do contribute important information to the body of knowledge concerning this distinctive loss of recurrent first trimester miscarriage from the perspective of men.

## 6 CONCLUSION

This valuable piece of research involved inquiry that generated knowledge and affirmed existing viewpoints regarding some of the experiences of men going through pregnancy loss. It is imperative that men's loss as a father is adequately recognized and acknowledged by clinicians and that adequate support such as the timely provision of appropriate information is made available to help men and their partners, eventually ending the stigma surrounding miscarriage. Men experiencing recurrent miscarriage run the risk of being doubly disenfranchised, with neither their unique experience as men nor their cumulative losses being recognized.

## AUTHOR CONTRIBUTIONS

Tommy Harty was involved in draft manuscript preparation, manuscript revision and interpretation of results. Maria Trench was involved in study conception and design, data collection, analysis and interpretation of results. Orla Keegan was involved in project supervision, study design, analysis and interpretation of results. Keelin O'Donoghue was involved in manuscript review and editing. Daniel Nuzum was involved in study conception and design, analysis and interpretation of results, manuscript review and editing. All authors reviewed the results and approved the final version of the manuscript.

## Data Availability

All relevant data are available within the paper and its Supporting Information Files.
